# Intravenously Injected Amyloid-β Peptide With Isomerized Asp7 and Phosphorylated Ser8 Residues Inhibits Cerebral β-Amyloidosis in AβPP/PS1 Transgenic Mice Model of Alzheimer’s Disease

**DOI:** 10.3389/fnins.2018.00518

**Published:** 2018-08-23

**Authors:** Sergey A. Kozin, Evgeny P. Barykin, Georgy B. Telegin, Alexander S. Chernov, Alexei A. Adzhubei, Sergey P. Radko, Vladimir A. Mitkevich, Alexander A. Makarov

**Affiliations:** ^1^Engelhardt Institute of Molecular Biology, Russian Academy of Sciences, Moscow, Russia; ^2^Pushchino Branch of Shemyakin-Ovchinnikov Institute of Bioorganic Chemistry, Russian Academy of Sciences, Moscow, Russia; ^3^Institute of Biomedical Chemistry, Moscow, Russia

**Keywords:** Alzheimer’s disease, amyloid-β peptide, zinc, isoaspartate, serine phosphorylation, transgenic mice, cerebral amyloidosis

## Abstract

Cerebral β-amyloidosis, an accumulation in the patient’s brain of aggregated amyloid-β (Aβ) peptides abnormally saturated by divalent biometal ions, is one of the hallmarks of Alzheimer’s disease (AD). Earlier, we found that exogenously administrated synthetic Aβ with isomerized Asp7 (isoD7-Aβ) induces Aβ fibrillar aggregation in the transgenic mice model of AD. IsoD7-Aβ molecules have been implied to act as seeds enforcing endogenous Aβ to undergo pathological aggregation through zinc-mediated interactions. On the basis of our findings on zinc-induced oligomerization of the metal-binding domain of various Aβ species, we hypothesize that upon phosphorylation of Ser8, isoD7-Aβ loses its ability to form zinc-bound oligomeric seeds. In this work, we found that (i) *in vitro* isoD7-Aβ with phosphorylated Ser8 (isoD7-pS8-Aβ) is less prone to spontaneous and zinc-induced aggregation in comparison with isoD7-Aβ and intact Aβ as shown by thioflavin T fluorimetry and dynamic light scattering data, and (ii) intravenous injections of isoD7-pS8-Aβ significantly slow down the progression of institutional β-amyloidosis in AβPP/PS1 transgenic mice as shown by the reduction of the congophilic amyloid plaques’ number in the hippocampus. The results support the role of the zinc-mediated oligomerization of Aβ species in the modulation of cerebral β-amyloidosis and demonstrate that isoD7-pS8-Aβ can serve as a potential molecular tool to block the aggregation of endogenous Aβ in AD.

## Introduction

The deposition of amyloid-β peptides (Aβ) as extracellular polymeric aggregates (so-called amyloid plaques) abnormally enriched with Zn, Cu, and Fe ions in specific brain regions is one of the hallmarks of Alzheimer’s disease (AD) (Cummings, 2004). The formation of brain amyloid plaques (cerebral β-amyloidosis) is Zn-dependent (Friedlich et al., 2004; Frederickson et al., 2005) and closely associated with neuronal loss and cognitive impairment (Musiek and Holtzman, 2015). In all mammals, Aβ binds zinc ions through its metal-binding domain formed by residues 1–16 (Aβ16) (Kozin et al., 2001; Zirah et al., 2006; Faller and Hureau, 2009; Tsvetkov et al., 2010; Istrate et al., 2012). This domain also contains the 11–14 segment responsible for zinc-induced dimerization of Aβ (Kozin et al., 2011), leading to the formation of stable Aβ aggregates with a beta-parallel arrangement of the monomers (Miller et al., 2010).

Endogenous Aβ is generated by the sequential cleavage of the amyloid precursor protein by β- and γ-secretase, producing peptides with lengths that vary from 37 to 43 amino acids (Benilova et al., 2012). It is a normal component of biological fluids of humans and other mammals at picomolar concentration levels (Masters and Selkoe, 2012). The 42-mer variant (Aβ42) is one of the most aggregation-prone Aβ isoforms and is found as the main component of amyloid plaques (Finder and Glockshuber, 2007). In transgenic animal models of AD, pathological conversion of the intact Aβ from its monomeric state into fibrillar supramolecular aggregates can be induced by exogenous injections of chemically or structurally modified Aβ species present in the AD amyloid plaques (Meyer-Luehmann et al., 2006). The origins of such Aβ modifications are ostensibly linked to AD-associated stresses, e.g., aging, neurotrauma, and neuroinflammation (Barykin et al., 2017).

Recently, we have discovered that the Aβ42 species with isomerized Asp7 (isoD7-Aβ42), which is one of the most abundant age-related Aβ species within amyloid plaques (Roher et al., 1993a,b; Shimizu et al., 2005; Moro et al., 2018), is to date the only known synthetic exogenous trigger of extensive amyloid plaque formation in AD models (Kozin et al., 2013; Kulikova et al., 2016). Even though isoD7-Aβ42 and Aβ42 have practically the same capacity for spontaneous aggregation *in vitro* (Fukuda et al., 1999; Kozin et al., 2013), isoD7-Aβ42 is much more toxic for neuronal cells than the intact Aβ42 (Mitkevich et al., 2013). The metal-binding domain of isoD7-Aβ42 was earlier shown to be extremely susceptible to zinc-induced oligomerization (Tsvetkov et al., 2008; Istrate et al., 2016), which strongly supports the importance of this process in AD cerebral β-amyloidosis (Kulikova et al., 2015; Kozin et al., 2016).

It was suggested that the prominent amyloidogenic features of isoD7-Aβ42 in comparison with the native Aβ42 (Kozin et al., 2013) are linked to the potential capacity of isoD7-Aβ42 to more readily generate zinc-induced oligomers relative to Aβ42 (Kulikova et al., 2015; Kozin et al., 2016; Kugaevskaya et al., 2018). This suggestion originated from the studies on interactions between zinc ions and the metal-binding domains of native Aβ and of the various naturally occurring Aβ species. Here, we focus on Aβ with the following post-translational modifications: isomerization of Asp 7 (isoD7-Aβ) and phosphorylation of Ser 8 (Tsvetkov et al., 2008; Kulikova et al., 2014; Istrate et al., 2016). It is worth noting that while the presence of isoD7-Aβ in amyloid plaques has been described in 1993 (Roher et al., 1993a), Aβ species with phosphorylated Ser8 (pS8-Aβ) have been found in AD patients relatively recently (Kumar et al., 2011; Rijal Upadhaya et al., 2014). The accumulation of isoD7-Aβ in amyloid plaques can be associated with the processes of spontaneous protein aging (Moro et al., 2018), while the formation of pS8-Aβ was attributed to the ecto-PKA activity (Kumar et al., 2011). Both isoforms of Aβ were suggested to play a role in AD pathogenesis (Kozin et al., 2016; Jamasbi et al., 2017; Kugaevskaya et al., 2018; Zatsepina et al., 2018).

Notably, Ser8 phosphorylation causes Aβ16 with such modification (pS8-Aβ16) to form zinc-bound homodimers but prevents it from further oligomerization in the presence of zinc ions (Kulikova et al., 2014). Moreover, insights into the molecular mechanism of zinc-mediated Aβ16 oligomerization indicate that pS8-Aβ16 can form zinc-driven heterodimers with Aβ species containing the primary zinc recognition site 11EVHH14, and such heterodimers might lack the ability to oligomerize (Istrate et al., 2016; Mezentsev et al., 2016; Polshakov et al., 2017). Based on the above, we have hypothesized that incorporating phosphorylated Ser8 into isoD7-Aβ42 resulting in the peptide with both isomerized Asp7 and phosphorylated Ser8 residues (isoD7-pS8-Aβ42) could safeguard such species from zinc-induced oligomerization *in vitro* and *in vivo*. To test this hypothesis, in the present study we have examined by thioflavin T (ThT) fluorometry and dynamic light scattering the ability of isoD7-pS8-Aβ42 to undergo spontaneous and zinc-induced aggregation *in vitro*, in comparison with synthetic peptides Aβ42 and isoD7-Aβ42 respectively. Subsequently, we have studied the effect of intravenous injections of isoD7-pS8-Aβ42 on the modulation of cerebral amyloidosis in the AβPP/PS1 transgenic mice model of AD.

## Materials and Methods

### Host Mice

Animals of mouse strain B6C3-Tg(APPswe,PSEN1dE9)85Dbo/j (stock number 004462), received from the Jackson Laboratory (JAX, ME, United States), were used in the study. Pedigree animal breeding and control genotyping procedures were conducted in compliance with the manufacturer’s recommendations. Laboratory animals were produced and housed under specific pathogen-free conditions at the AAALAC-accredited Animal Breeding Facility, Branch of the Shemyakin and Ovchinnikov Institute of Bioorganic Chemistry, Russian Academy of Sciences (Pushchino, Moscow Region, Russia). In the course of experiments, the animals were kept under standard housing conditions in the barrier area according to the Institutional Animal Care and Use Program and the IACUC-approved Study Protocol. The experimental procedures were approved by the local Animal Care and Use Committee (Reg#126/15 from March 31, 2015).

### Reagents

All chemicals and solvents used throughout this study were of HPLC-grade or better and were obtained from Sigma-Aldrich (St. Louis, MO, United States) unless otherwise stated. Synthetic peptides (purity >98% checked by RP-HPLC) [H2N]-DAEFRHDSGYEVHHQKLVFFAEDVGSNKGAIIGLMVGGVVIA-[COOH] (Aβ42), [H_2_N]-DAEFRH[isoD]SGYEVHHQKLVFFAEDVGSNKGAIIGLMVGGVVIA-[COOH] (isoD7-Aβ42), and [H_2_N]-DAEFRH[isoD][pS]GYEVHHQKLVFFAEDVGSNKGAIIGLMV GGVVIA-[COOH] (isoD7-pS8-Aβ42) were purchased from Biopeptide (San Diego, CA, United States). Amino acid sequences of the peptides were confirmed on an ultrahigh-resolution Fourier transform ion cyclotron resonance mass spectrometer 7T Apex Qe (Bruker Daltonics, Billerica, MA, United States) by using a *de novo* sequencing approach based on a collision-induced dissociation (CID) fragmentation technique as described in an earlier work (Indeykina et al., 2011).

### Preparation of Aβ Peptide Samples for Aggregation Tests

To prepare monomeric solutions of Aβ42, isoD7-Aβ42, and isoD7-pS8-Aβ42, the peptides were treated with hexafluoroisopropanol, dried, and dissolved in 10 mM NaOH at a concentration of 0.5 mM. The solutions of Aβ isoforms in 10 mM NaOH were adjusted with 100 mM HEPES-buffer (pH 5.0) to pH 7.4 and subjected to centrifugation (10 min, 16,000 *g*, 4°C) to remove insoluble peptide aggregates. Peptides in the supernatant were quantified spectrophotometrically based on the molar extinction coefficient 𝜖_280_ = 1490 M^-1^cm^-1^ (Jan et al., 2010) and diluted with appropriate buffers to provide 30 μM Aβ solutions in buffer containing 10 mM HEPES (pH 7.4) and 150 mM NaCl (further referred to as buffer H). Aβ solutions were kept on ice before further use. To prepare zinc-induced Aβ aggregates, an aliquot of 30 μM Aβ solution was mixed with buffer H supplemented with 300 μM ZnCl_2_ to provide the final Aβ concentration of 25 μM and zinc/Aβ molar ratio of two. The mixture was used for dynamic light scattering (DLS) measurements after 20 min incubation at room temperature.

### Dynamic Light Scattering

DLS measurements were carried out on a Zetasizer Nano ZS system (Malvern Instruments Ltd., Malvern, United Kingdom) at 25°C. The system is able to measure particle sizes ranging from 0.6 nm to 10 μm. Autocorrelation functions were collected at specified time intervals with acquisition times of 120 s per measurement and converted by the instrument software into particle size distributions, assuming the viscosity and the refractive index of the buffer to be equal to those of water (0.89 cP and 1.333, respectively). The instrument software provides the particle size distribution and the average particle diameter, approximating a heterogeneous population of zinc-induced Aβ aggregates by a population of spherical particles with identical distributions of diffusion coefficients. Consequently, the characteristic size of Aβ aggregates is expressed in terms of the average “diameter.” Number particle size distributions were used to calculate average diameters.

### Fluorimetry

Fluorescence measurements were carried out on the Infinite M200 PRO microplate reader (TECAN, Switzerland) using Corning 96-well microplates. The excitation and emission wavelengths were set to 450 and 482 nm, respectively. To test the self-aggregation of Aβ peptide isoforms, 100 μl aliquots of Aβ solutions (peptide concentration: 30 μM) were mixed in wells with 20 μl of the ThT solution in buffer H (ThT concentration: 150 μM), followed by incubation at 37°C with constant agitation. The final Aβ and ThT concentrations were 25 μM. The fluorescence measurements were started immediately after the preparation of Aβ/ThT mixtures. All measurements were made in triplicates. The values of fluorescence in the wells containing buffer alone were subtracted from those in wells containing ThT. The relative changes of ThT fluorescence intensity were calculated as (F–F0)/F0, where F and F0 are the fluorescence intensities of ThT in the presence and absence of Aβ peptides, respectively.

### Synthetic isoD7-pS8-Aβ42 Preparations for Injections

Two-thousand micrograms of isoD7-pS8-Aβ42 were dissolved in 2,000 μl of sterile physiological saline (PS), and the prepared solution was filtered through a 0.22 μm filter (Millex-GV, Millipore), aliquoted to 125 μl and frozen. For injection, one aliquot was thawed, and sterile PS was added to obtain 1,500 μl of solution with a peptide concentration of 0.08333 μg per μl (“administration solution”). Then, 150 μl of the “administration solution” were withdrawn and 125 μl of this solution were injected into one animal. Thus, with a single injection into the mouse blood, 10 μg of the peptide were injected. Each injection sample was prepared immediately before its introduction to the animals.

### Intravenous Injections

Retro-orbital injections in female mice were performed according to Yardeni et al. (2011). Mice received one intravenous injection with 1-month intervals between injections. The compositions of injections for each group of mice are presented in **Table 1**. The mice were assigned to the various groups randomly.

**Table 1:** | Suppression of congophilic amyloid plaque formation in the brain of B6C3-Tg(APPswe,PSEN1dE9)85Dbo/J transgenic mice by intravenous injections of isoD7-pS8-Aβ42.

Transgenic mice (females)				Injection	Brain sections	Number of congophilic amyloid plaques per section	Statistical significance

Group name	Number of animals	Age at first injection (months)	Age at sacrifice (months)	Administered compound/total number of injections	Total number	In regions CA1, CA2, CA3, and the dentate gyrus of the hippocampus (mean ± SEM)	vs. Control
Control	4	-	8	PS (125 μl)/6	40	28.7 ± 4.6	-
IsoD7-pS8-Aβ42	7	2	8	Synthetic isoD7-pS8-Aβ42 (10 μg in 125 μl of PS)/6	70	7.4 ± 2.8	*p* < 0.001


### Histology and Immunohistochemistry

The euthanasia procedure was applied to 8-month-old mice. Mouse euthanasia was carried out using CO_2_ according to the IACUC-approved protocol with the use of automated CO_2_-box (Bioscape, Germany). Mice were transcardially perfused with 50 mL of PBS, followed by 50 mL of 4% paraformaldehyde (PFA). Mouse brains were fixed in 10% formalin. Processing for paraffin embedding was scheduled as follows: 75% ethanol overnight, 96% ethanol 5 min, 96% ethanol 10 min, 100% ethanol 10 min (two changes), ethanol–chloroform (1:1) 30 min, and chloroform overnight. Paraffin embedding was performed at 60°C for 3 h (three changes). The embedding of tissues into paraffin blocks was performed using a Leica EG1160 device. Serial brain sections (8 μm) were cut using a Leica RM2265 microtome mounted onto slides. For deparaffinization, hydration, and staining of the sections, the following steps were performed. Slides were consistently placed in xylene with three changes (10 min each), 96% ethanol (5 min), 90% ethanol (2 min), 75% ethanol (2 min), H_2_O with three changes (5 min each), Congo red solution (5 min), potassium alkali solution, and water. The Immu-Mount medium (Thermo Scientific) was used for mounting.

Immunostaining was carried out as described elsewhere (Kozin et al., 2013). Briefly, sections were deparaffinized and after antigen retrieval by microwaving in citrate buffer washed in PBS and blocked with 10% goat serum in 0.04% Tween 20 in PBS (T-PBS). Sections were incubated with the primary antibody for 2 h at room temperature, washed thrice in T-PBS, and incubated with the secondary antibody for 1 h at room temperature followed by washing in T-PBS. The mouse antihuman Aβ monoclonal antibodies 6E10 (Covance, Dallas, TX, United States) diluted in the block solution 1:1000 were used as the primary antibodies, and Alexa Fluor 488 goat anti-mouse antibodies (Invitrogen, Grand Island, NY, United States) were used as the secondary antibodies for immunofluorescence staining (dilution 1:1000 in T-PBS). The images were captured with a Leica DFS490 digital camera (Leica Microsystems, Wetzlar, Germany) at 100× magnification using a Leica DMI 4000 fluorescent microscope (Leica Microsystems, Wetzlar, Germany).

### Quantitative Assessment of Cerebral β-Amyloidosis

The sections spanning the brain from 0.48 to 1.92 mm relative to the midline in lateral stereotaxic coordinates (Franklin and Paxinos, 2008) were used to quantify the congophilic amyloid plaques in the dentate gyrus and the CA1, CA2, and CA3 regions of the hippocampus. Every 15th section was analyzed, yielding 10 sections per animal. Amyloid plaques were identified by Congo red staining and manually counted as described previously (Kozin et al., 2013; Kulikova et al., 2016) using a Zeiss Axiovert 200 M microscope with 10×, 20×, and 40× objectives (Carl Zeiss, Oberkochen, Germany), with examination under a bright field and between crossed polarizers. Amyloid plaques of all sizes were accepted for counting if they were visible and met the following requirements: red coloring under the bright field and green birefringence in polarized light. Analyses were undertaken by two researchers independently (EPB and SAK). To determine the reproducibility of the plaque counts, an intraclass correlation (ICC) was calculated yielding a good interrater reliability between the two researchers (ICC > 0.85). For each group of mice, the average values (±SEM) of the number of plaques per section were calculated.

### Statistical Methods Used for Data Analysis

Data were presented as means of at least three independent experiments ±SEM. The Shapiro-Wilk test was used to check the normality of the distribution. The Mann–Whitney test was used for a pairwise comparison between examined groups. The significance level applied was 99.9% (*p* < 0.001). Statistical analysis was performed using STATISTICA 8.0 (StatSoft, Inc., Tulsa, OK, United States).

## Results

### *In vitro* Spontaneous and Zinc-Induced Aggregation Behavior of isoD7-pS8-Aβ42 Significantly Differs From That of Aβ42 and isoD7-Aβ42

**FIGURE 1:**
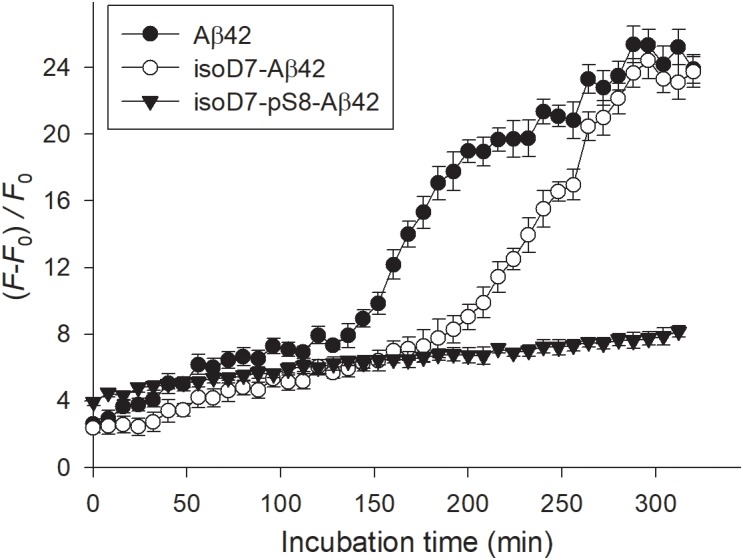
Relative changes of thioflavin T (ThT) fluorescence in the solutions of Aβ isoforms with incubation time. The Aβ isoforms studied are indicated in the insert. Peptides were incubated at 37°C with agitation. Peptide concentration: 25 μM; peptide/ThT molar ratio: 1. Buffer: 10 mM HEPES (pH 7.4), 150 mM NaCl. Mean values and standard errors for triplicate measurements are shown.

ThT is known to fluoresce upon binding to amyloid aggregates and the increase of ThT fluorescence is widely used as a qualitative measure of the aggregates’ β-sheet content. As seen from **Figure 1**, the incubation of Aβ42 solutions at 37°C with constant agitation leads to the increasing occurrence of β-sheet-rich Aβ42 aggregates. The aggregation is completed in about 5 h of incubation, when the values of ThT fluorescence apparently approach a plateau (**Figure 1**). The isomerization of D7 residue notably alters the kinetics of aggregation. Though the extent of aggregation for both Aβ42 and isoD7-Aβ42 peptides appears to be similar, a rapid rise of ThT fluorescence was observed later in the course of aggregation for the isoD7-Aβ42 isoform (within the incubation time interval of approximately 200 to 290 min) in comparison with that for Aβ42 (approximately between 130 and 230 min of incubation, **Figure 1**). This observation suggests that the isomerization of D7 residue alters the aggregation kinetics for this Aβ isoform by slowing the formation of isoD7-Aβ42 aggregates characterized by the high β-sheet content. The occurrence of the second modification – the phosphorylation of Ser8 residue – further reduces the ability of Aβ peptides to form β-sheet-rich aggregates, as exemplified by the absence of a substantial increase of ThT fluorescence observed for the isoD7-pS8-Aβ42 isoform within the 5 h incubation interval tested (**Figure 1**). However, the initial values of ThT fluorescence in the case of isoD7-pS8-Aβ42 were found to lie above those for Aβ42 and isoD7-Aβ42 peptides. This suggests either a higher initial degree of aggregation for the isoD7-pS8-Aβ42 isoform or a different ability to bind ThT molecules, compared to Aβ42 and isoD7-Aβ42 peptides.

**FIGURE 2:**
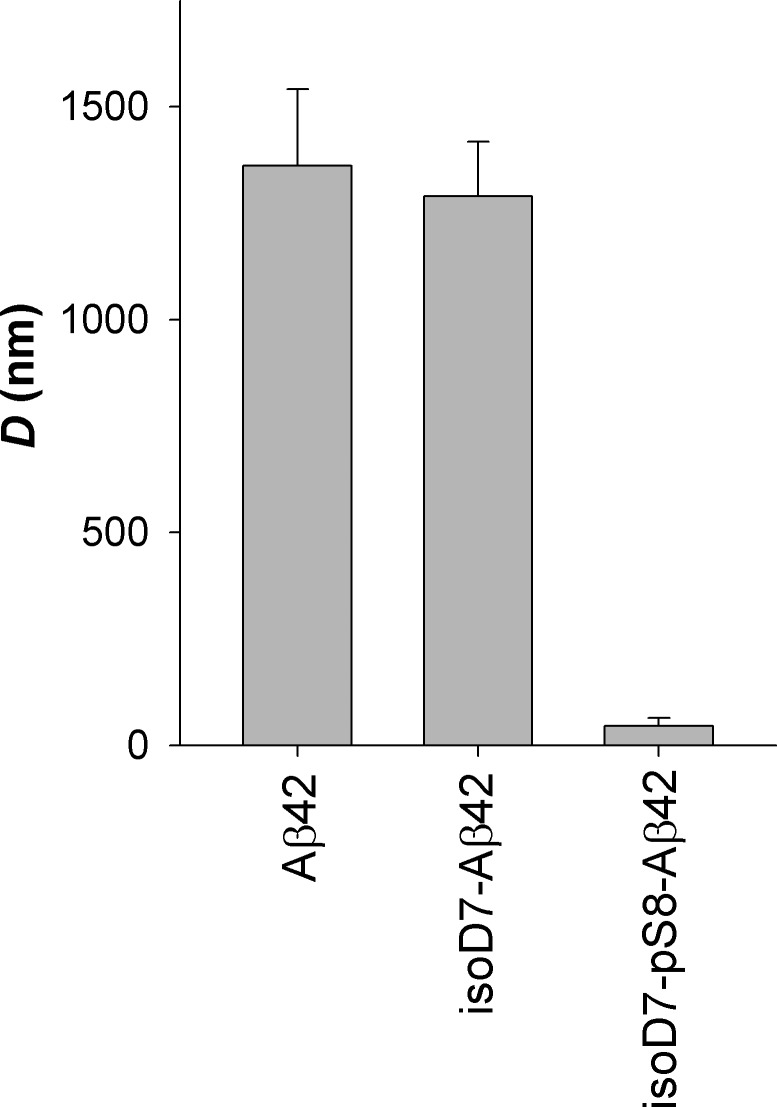
Characteristic diameter of zinc-induced aggregates for the studied Aβ isoforms. DLS measurements were carried out within 20 min after the addition of zinc to Aβ solutions. Peptide concentration: 25 μM; zinc/peptide molar ratio: 2. Buffer: 10 mM HEPES (pH 7.4), 150 mM NaCl. Mean values and standard deviations for three independent measurements are shown.

Using DLS, we measured the characteristic diameter of zinc-induced aggregates of Aβ42, isoD7-Aβ42, and isoD7-pS8-Aβ42 isoforms. The corresponding representative size distributions are presented in **Supplementary Figure S1** and the mean values of the characteristic diameters in **Figure 2**. Prior to zinc addition, only aggregates 15–25 nm in size was detected. No statistically significant differences between the sizes of the preexisting aggregate were found for the Aβ isoforms under study (data not shown). After 20 min of incubation with Zn^2+^ (the incubation time is due to the data presented in **Supplementary Figure S2**), the characteristic diameter of isoD7-pS8-Aβ42 aggregates was found to be remarkably different (45 ± 19 nm) from the diameters of Aβ42 and isoD7-Aβ42 aggregates that were statistically indistinguishable and equal to 1362 ± 180 nm and 1290 ± 128 nm, respectively (**Figure 2**). This observation suggests that the isoD7-pS8-Aβ42 isoform is substantially less susceptible to zinc-triggered aggregation, compared to Aβ42 and isoD7-Aβ42 isoforms. It should be noted that we observed no discernable changes in the characteristic size of Aβ oligomers for the peptide isoforms studied when incubating 25 μM Aβ solutions for 20 min under quiescent conditions at room temperature in the absence of zinc ions.

### Intravenous Injections of isoD7-pS8-Aβ42 Decrease the Amyloid Burden in Transgenic Mice

We investigated the ability of synthetic isoD7-pS8-Aβ42 peptide to reduce cerebral β-amyloidosis in the APP/PS1 doubly transgenic mouse model of AD. These mice demonstrated cognitive features of AD-like pathology and possessed significant amounts of dense-core congophilic amyloid plaques starting from 4 to 6 months age regardless of sex (Borchelt et al., 1997; Garcia-Alloza et al., 2006). The experimental groups included female animals subjected to intravenous injections of isoD7-pS8-Aβ42 (10 μg in 125 μl of PS) starting from the age of 2 months. After serial inoculations (at 1-month intervals) with the peptide, the host mice were sacrificed at the age of 8 months. Female mice injected with PS (125 μl) were used as control.

**FIGURE 3:**
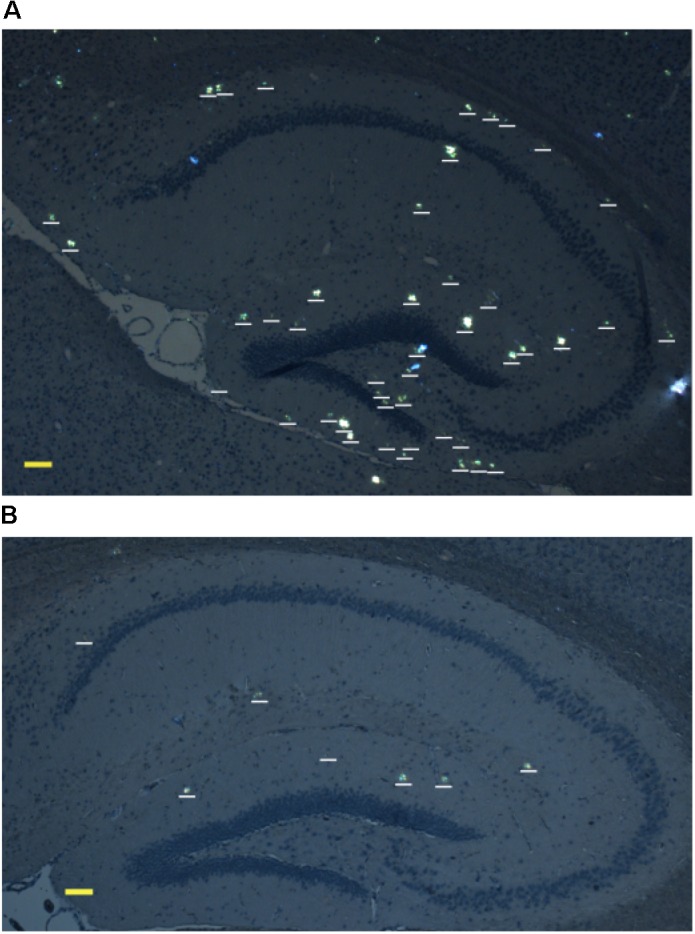
Representative polarized light micrographs of brain sections through the hippocampus for 8-month-old B6C3-Tg(APPswe, PSEN1dE9)85Dbo/j transgenic mice intravenously injected with sterile PS **(A)** or synthetic isoD7-pS8-Aβ42 peptide **(B)**. Amyloid plaques are highlighted by white bars. Scale bars: **(A,B)** 100 μm.

The brains were extracted, and sagittal brain sections (8 μm thick) were analyzed histochemically using Congo red staining (**Figure 3**). The hippocampus was chosen as the target region for manual counting of stained congophilic amyloid plaques by using bright-field and polarized light microscopy in the sections representing the brain layer located from 0.48 to 1.92 mm relative to the midline in lateral stereotaxic coordinates. The congophilic plaques found in the brains of all experimental animals were similar in terms of their localization and size distribution in the brain parenchyma (**Figure 3**). Additionally, congophilic amyloid plaques were characterized by immunohistochemical analysis, which showed the presence of Aβ (**Supplementary Figure S3**). However, quantitative analysis revealed a significantly lower number of congophilic amyloid plaques per section in the isoD7-pS8-Aβ42-inoculated 8 month-old transgenic mice (*p* < 0.01) compared to control littermates (**Table 1**).

## Discussion

The presence of dense congophilic amyloid plaques in the patient’s brain is a mandatory postmortem criterion for the final diagnosis of AD (Cummings, 2004). Studies in animal models of AD have shown that human Aβ and zinc ions are a prerequisite for the formation of amyloid plaques (Friedlich et al., 2004; Frederickson et al., 2005). However, neither intact Aβ itself nor its combination with zinc ions is amyloidogenic *in vivo* (Meyer-Luehmann et al., 2006). From transgenic mice studies, it has been suggested that structurally and/or chemically modified isoforms of Aβ can act as aggregation seeds, compelling endogenous molecules of Aβ to form soluble neurotoxic oligomers and insoluble extracellular aggregates (Meyer-Luehmann et al., 2006; Jucker and Walker, 2011). Indeed, isoD7-Aβ42 had been demonstrated to accelerate cerebral β-amyloidosis in transgenic mice upon serial intravenous injections (Kozin et al., 2013). Its amyloidogenicity was later attributed to the metal-binding domain isoD7-Aβ16 (Kulikova et al., 2016). The ability of peripherally derived Aβ species to directly contribute to AD pathogenesis has been recently confirmed in an independent study (Bu et al., 2017).

Based on the results of our previous studies (rev. in Kozin et al., 2016), we can hypothesize that the possible mechanism of isoD7-Aβ16 and isoD7-Aβ42 amyloidogenicity *in vivo* lie in their specific ability to form zinc-linked oligomers, which in the case of zinc-mediated interaction with endogenous Aβ molecules cause the latter to lose their native conformation. As a result, the modified Aβ molecules accumulate on the surface of zinc-dependent neurons in the form of amyloid plaques. The goal of this study was to test our hypothesis regarding the role of zinc-bound oligomers of isoD7-Aβ42 as aggregation seeds for endogenous Aβ in the transgenic model of AD. To achieve this, we have constructed an artificial peptide, isoD7-pS8-Aβ42, which in our opinion should block the formation of such zinc-induced oligomers, and as a consequence significantly slow down the formation of amyloid plaques in transgenic mice.

In contrast to Asp7 isomerization, Ser8 phosphorylation inhibits zinc-induced oligomerization of Aβ16 (Kulikova et al., 2014). In the present work, we have shown that the spontaneous aggregation (resulting in the formation of Aβ aggregates with high β-sheet content) of isoD7-pS8-Aβ42 is decreased in comparison with Aβ42 and isoD7-Aβ42 (**Figure 1**). This decrease in the propensity of isoD7-pS8-Aβ42 to undergo spontaneous aggregation is quite unusual in the light of previously obtained data showing that pS8-Aβ42 is much more prone to spontaneous aggregation than Aβ42 (Kumar et al., 2011). We have also found that zinc-induced oligomerization of isoD7-pS8-Aβ42 appears to be less prominent than that of the other peptides under study (**Figure 2**). This effect most likely appears due to the fact that isoD7-pS8-Aβ42 interacts with zinc ions similarly to pS8-Aβ16; i.e., after the formation of zinc-linked dimer through sites 11–14 of the interacting molecules, further zinc-dependent oligomerization of isoD7-pS8-Aβ42 is stopped because of the absence of the second interface on the surface of such a dimer (Istrate et al., 2016). Taken together, observations *in vitro* demonstrated a significant effect of the phosphorylation of Ser8 on the aggregation properties of isoD7-Aβ42.

Experiments on transgenic mice revealed an approximately fourfold reduction in the number of congophilic amyloid plaques in animals injected with isoD7-pS8-Aβ42 in comparison with the control littermates (**Figure 3** and **Table 1**). To the best of our knowledge, such an anti-amyloid effect has been shown for the first time for intravenously administered synthetic peptides. Earlier we showed that in the animal model of AD similar to the one used in this study, intravenous injections of isoD7-Aβ42 significantly accelerated the process of amyloidogenesis (Kozin et al., 2013). Overall, we have established that for isoD7-pS8-Aβ42, there is a direct correlation between its low zinc-mediated oligomerization *in vitro* and its ability to suppress cerebral β-amyloidosis *in vivo*. Thus, Ser8 phosphorylation substantially neutralizes the pathogenic features of isoD7-Aβ42. These data support the role of modified Aβ peptides as key factors regulating cerebral amyloidosis in AD.

## Author Contributions

SK, EB, GT, AC, AA, and SR performed the experiments and analyzed the data. SK, VM, and AM conceived the experiments and coordinated the project. SK and AA wrote the manuscript. All authors read and edited the manuscript.

## Conflict of Interest Statement

The authors declare that the research was conducted in the absence of any commercial or financial relationships that could be construed as a potential conflict of interest.
