# Supplementary material for: Intravenously Injected Amyloid-β Peptide With Isomerized Asp7 and Phosphorylated Ser8 Residues Inhibits Cerebral β-Amyloidosis in AβPP/PS1 Transgenic Mice Model of Alzheimer’s Disease
Source: Front Neurosci. 2018 Aug 23;12:518. doi: 10.3389/fnins.2018.00518 (PMC6119768; doi:10.3389/fnins.2018.00518)
Supplement: Supplementary file 1 [file Presentation_1.pdf]

Supplementary Material

Intravenously injected amyloid-β peptide with isomerized Asp7 and phosphorylated Ser8 residues inhibits cerebral β-amyloidosis in AβPP/PS1 transgenic mice model of Alzheimer's disease

Sergey A. Kozin[1*], Evgeny P. Barykin[1], Georgy B. Telegin[2], Alexander S. Chernov[2], Alexei A. Adzhubei[1], Sergey P. Radko[1,3], Vladimir A. Mitkevich[1], Alexander A. Makarov[1]

[1]Engelhardt Institute of Molecular Biology, Russian Academy of Sciences, Moscow, Russia
[2]Puschchino Branch of Shemyakin-Ovchinnkiov Institute of Bioorganic Chemistry, Russian Academy of Sciences, Moscow, Russia
[3]Institute of Biomedical Chemistry, Moscow, Russia

*** Correspondence:**
Dr. Sergey A. Kozin
kozinsa@gmail.com

Panel A

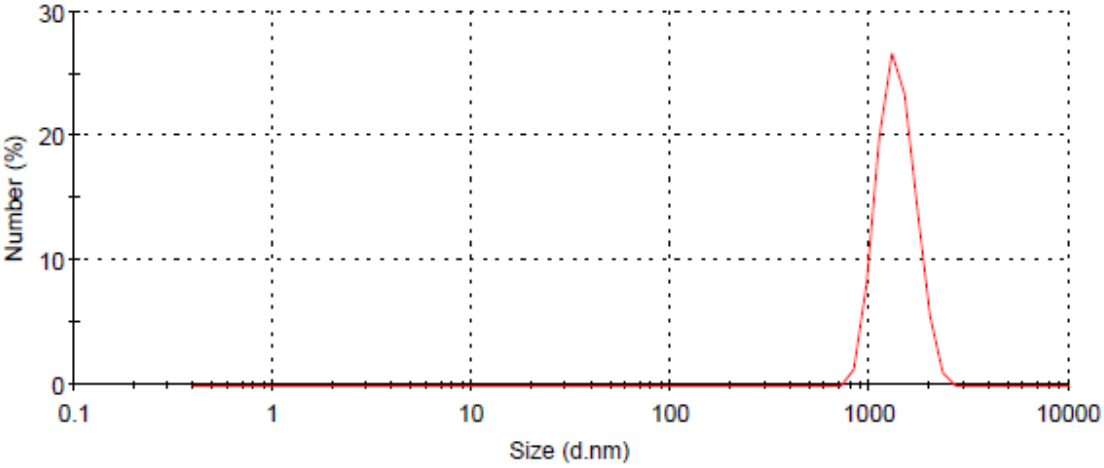

Panel B

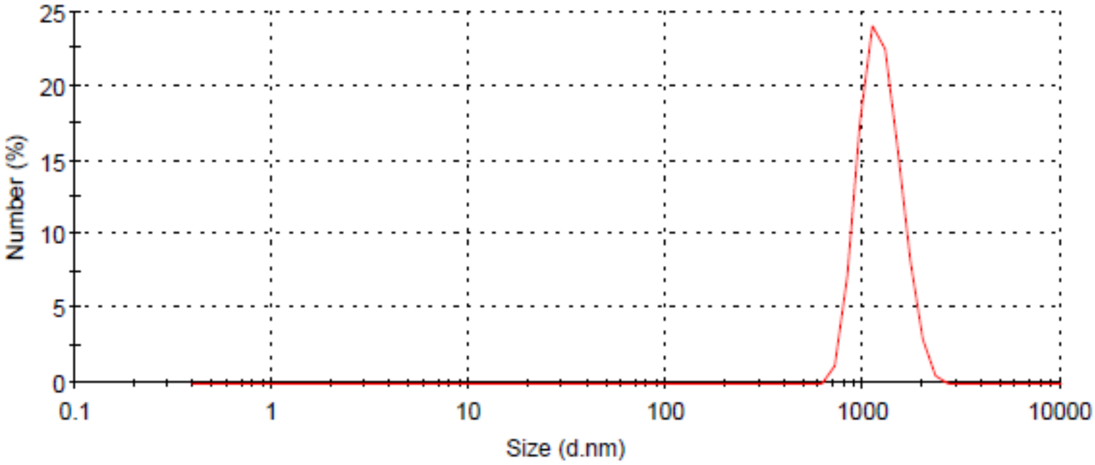

Panel C

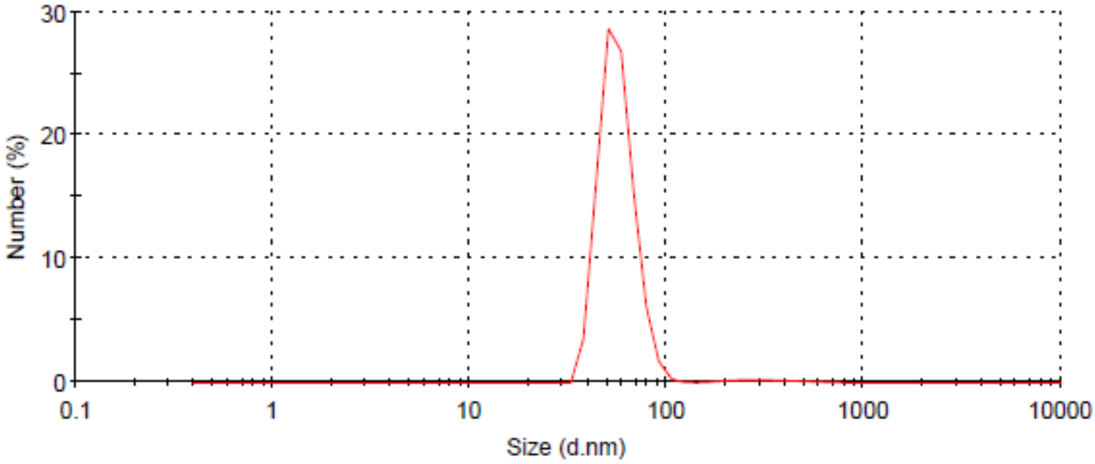

Fig. S1. The representative results of DLS measurements. The graphic output of the Zetasizer Nano ZS apparatus: size distributions by number (fraction of particles of a given diameter, %) for zinc-induced aggregates of Aβ42, isoD7-Aβ42, and isoD7-pS8-Aβ42 (panels A, B, and C, respectively).

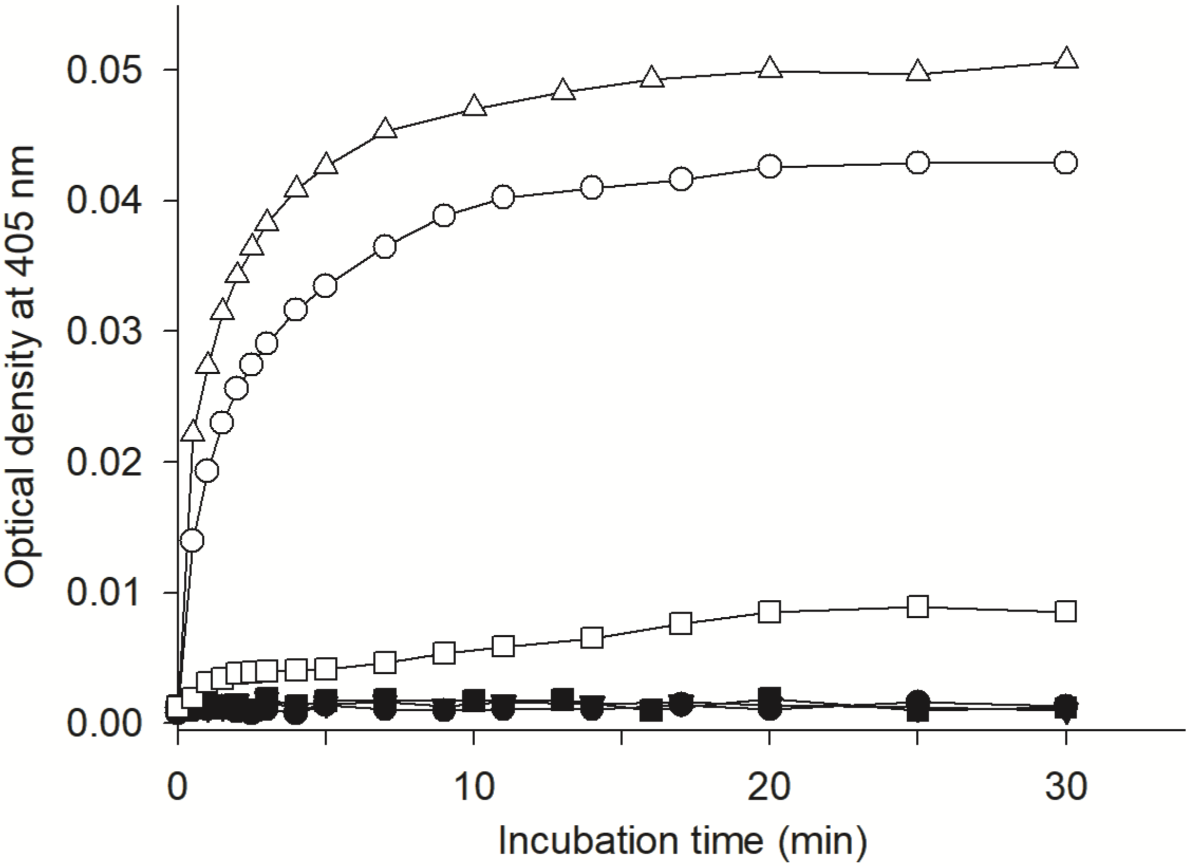

Fig. S2. The representative dependencies of Aβ solution turbidity (optical density at 405 nm) on the incubation time in the presence (open symbols) and the absence (closed symbols) of zinc ions. Triangles – Aβ42; circles – isoD7-Aβ42; rectangles – isoD7-pS8-Aβ42. Peptide concentration – 25 µM; peptide/zinc molar ratio = 1:2. Buffer H (10 mM HEPES, pH 7.4, 150 mM NaCl). A peptide/zinc mixture was placed in a BRAND UV microcuvetter (BRAND GMBH, Germany) and its turbidity was monitored in time at room temperature using an Agilent 8453E spectrophotometer (Agilent Technologies, USA). Turbidity measurements were started in 0.5 min after mixing 125 µL of the 30-µM Aβ solution with 25 µL of 300-µM solution of $ZnCl_2$ in buffer H. The initial (zero time) points were measured in the absence of zinc ions, using 25-µM Aβ solutions.

Fig. S3. - A

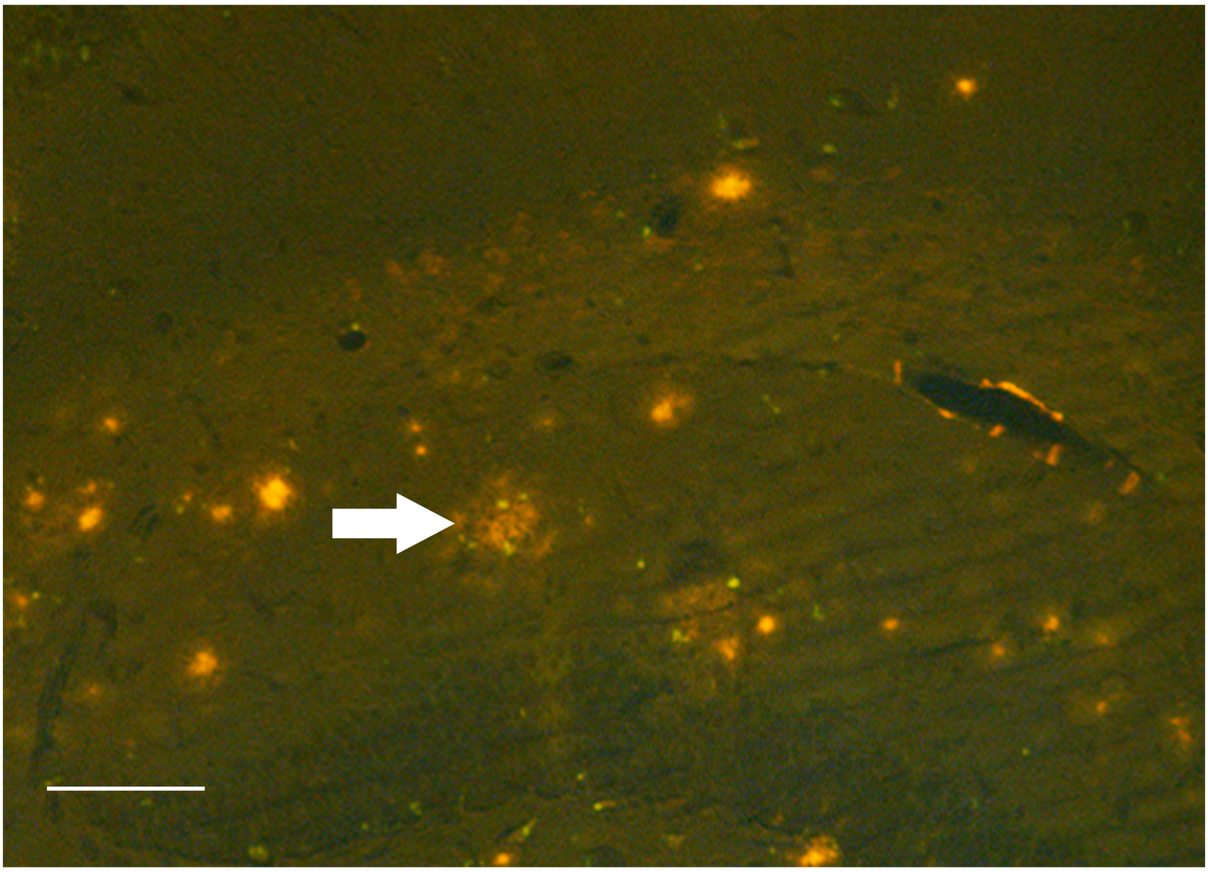

Fig. S3. - B

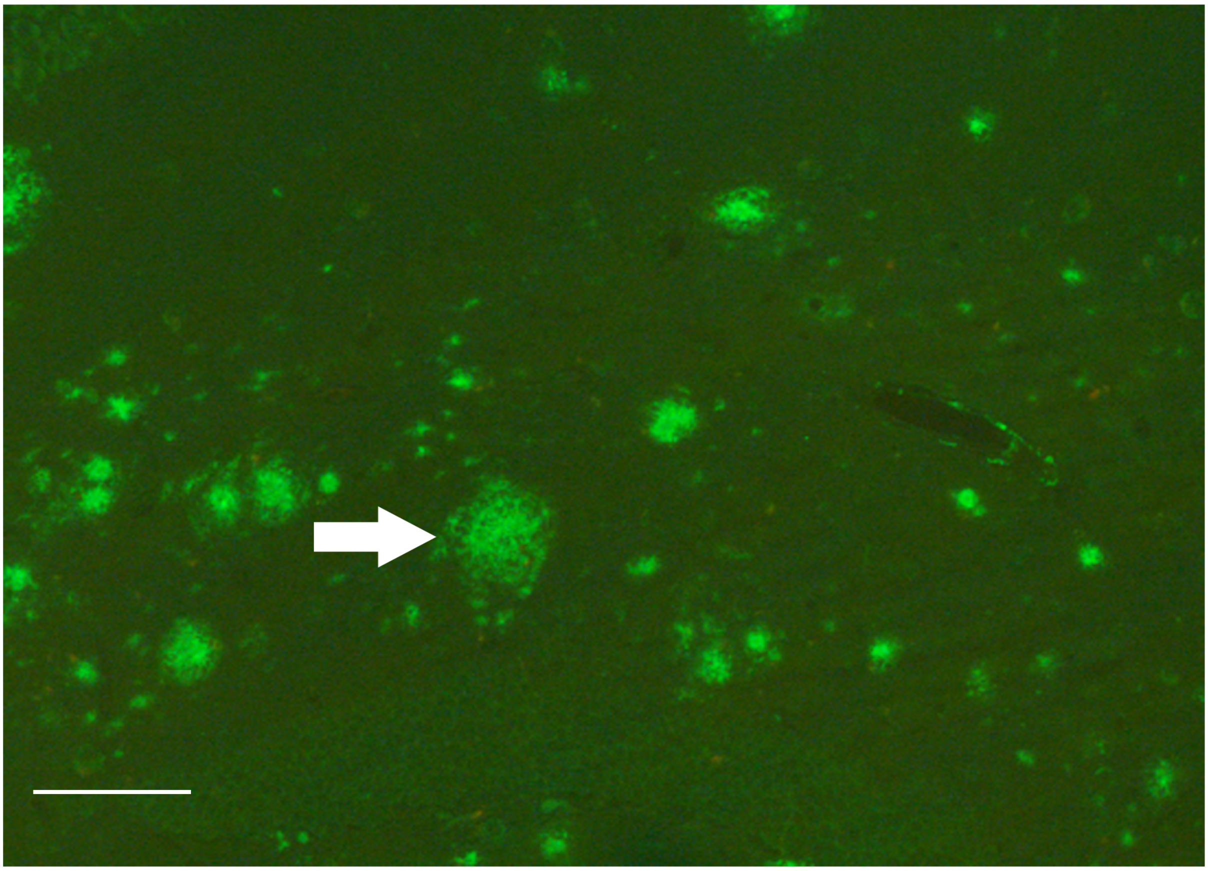

Fig. S3. - C

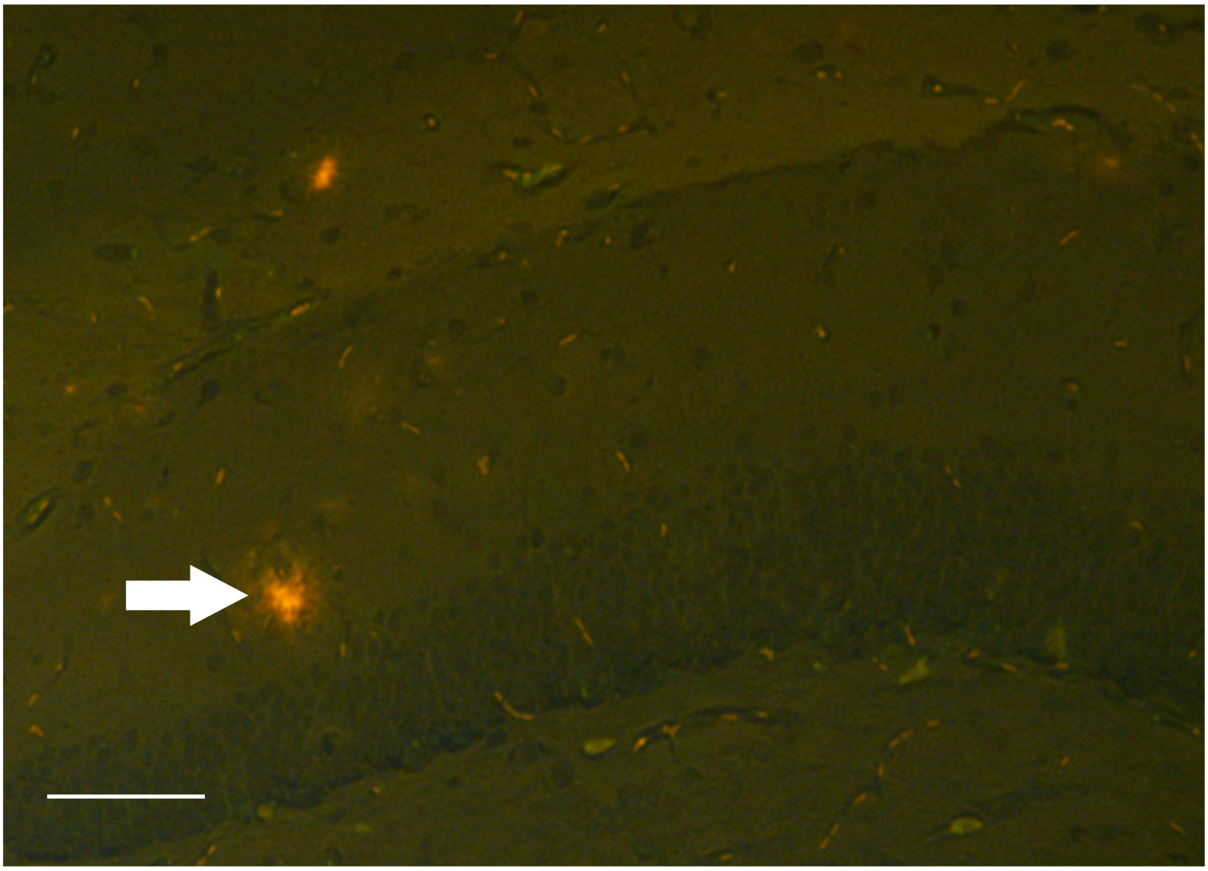

Fig. S3. - D

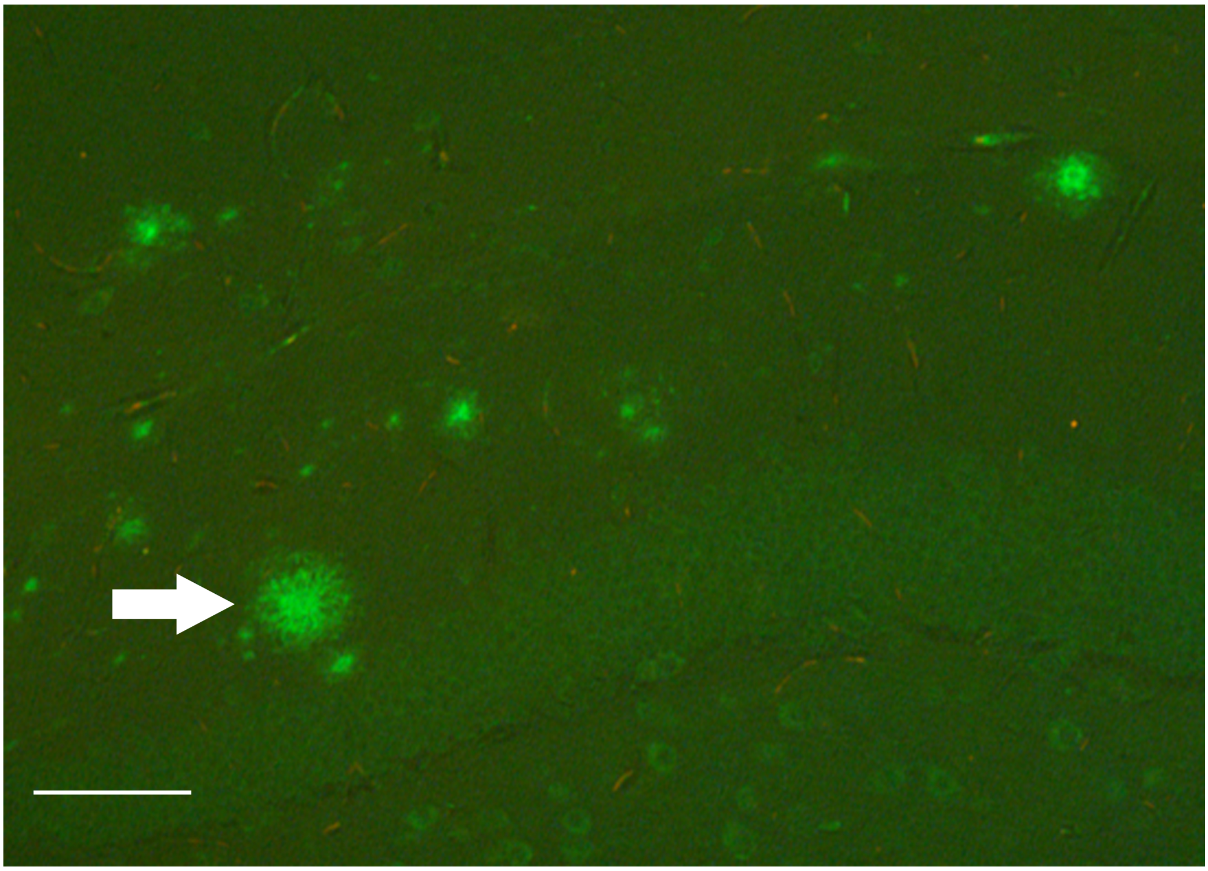

Fig. S3. Representative fluorescent micrographs of brain sections through the hippocampus for 8-month-old B6C3-Tg(APPswe,PSEN1dE9)85Dbo/j transgenic mice intravenously injected with sterile PS (**A, B**) or synthetic isoD7-pS8-Aβ42 peptide (**C, D**). Counterstaining of adjacent sections of the brain in the dentate gyrus of the hippocampus by Congo Red dye (**A, C**), and immunohistochemical staining by specific antibodies to Aβ (**B, D**). Selected amyloid inclusions are indicated by white arrows. Scale bars: (**A, B, C, D**) 100 µm.
